# Blood biomarker changes and relationships after low dose oral ketamine treatment for post-traumatic stress disorder (PTSD)

**DOI:** 10.1007/s00213-026-07043-6

**Published:** 2026-03-16

**Authors:** Bonnie L. Quigley, Emerald Orr, Sophie Kafka, Maryam Hajishafiee, Ana P. Bouças, Nathan Wellington, Megan Dutton, Monique Jones, Fiona Randall, Jim Lagopoulos, Adem T. Can, Daniel F. Hermens

**Affiliations:** 1 National PTSD Research Centre at the Thompson Institute, University of the Sunshine Coast, Birtinya, QLD 4575 Australia; 2 Centre for Bioinnovation, University of the Sunshine Coast, Sippy Downs, QLD 4556 Australia; 3 Clinical Trials Centre, University of the Sunshine Coast, Brisbane, QLD 4101 Australia; 4 Thompson Brain and Mind Healthcare, Maroochydore, QLD 4558 Australia; 5 School of Medicine and Psychology, ANU College of Science and Medicine, Canberra, ACT 2600 Australia; 6 Thompson Institute, University of the Sunshine Coast, 12 Innovation Parkway, Birtinya, QLD 4575 Australia

**Keywords:** BDNF, VEGF-A, cytokines, FKBP51, serotonin, biomarker, ketamine, PTSD

## Abstract

**Rational:**

Ketamine has been investigated as a treatment alternative for PTSD for the last 20 years, yet there have been limited reports of biological changes or biomarker characterisation related to treatment.

**Objectives:**

To address this significant gap, this study analysed blood samples from 25 participants with PTSD who took part in an open-label 6-week trial of low dose oral ketamine treatment.

**Methods:**

Serum and plasma samples were quantified before and after ketamine treatment for brain-derived neurotrophic factor (BDNF), vascular endothelial growth factor A (VEGF-A), serotonin, FK506 binding protein 51 (FKBP51) and a panel of cytokines (interleukin (IL)-1β, IL-2, IL-4, IL-6, IL-12p70, IL-17 A and tumour necrosis factor alpha (TNFα)).

**Results:**

Analysis of BDNF and VEGF-A levels from serum detected a significant positive correlation between the two biomarkers and a small but statistically significant decrease in both measures after ketamine treatment. This novel finding reinforces theories that ketamine’s effects may rely on a reciprocal interaction between BDNF and VEGF-A, offering potential insights into a biological mechanism underpinning PTSD symptom reduction. Additionally, the analysis of FKBP51 and serotonin revealed novel relationships between these biomarkers and clinical scales, before and after ketamine treatment. Finally, significant changes or relationships involving the immune cytokines were not detected, possibly because half the participants presented with low-grade inflammation while the other half did not.

**Conclusions:**

This study represents a much-needed broad analysis of blood biomarkers before and after ketamine treatment for PTSD and reveals important biological changes and relationships related to this treatment.

**Supplementary Information:**

The online version contains supplementary material available at https://doi.org/10.1007/s00213-026-07043-6.

## Introduction

Trauma is relatively common, with a consensus indicating more than 70% of people will experience a traumatic event in their lifetime (Benjet et al. 2016). From these experiences, ~ 10% of people will go on to develop post-traumatic stress disorder (PTSD), characterised by re-experiencing, avoidance, increased negative affect and hyperarousal symptoms (American Psychiatric Association 2013; Australian Bureau of Statistics 2020-21). In contemporary clinical settings, PTSD treatment often includes a combination of psychological and pharmacological interventions (Green 2013; Thomas and Stein 2017). Exposure-based psychotherapies have the largest evidence base and aim to reduce symptoms by encouraging adaptive associations with painful memories, rendering the trauma tolerable (López-Ojeda and Hurley 2022). However, treatment response rates from current exposure-based protocols report only 46–60% of patients achieve recovery with these treatments (Klaeth et al. 2024). Antidepressants, particularly selective serotonin reuptake inhibitors (SSRIs), are the primary treatment medication for PTSD in pharmacotherapy (Asnis et al. 2004). Two SSRIs are FDA-approved for PTSD treatment, paroxetine and sertraline, and response rates to these drugs rarely exceed 60%, with less than 30% of patients achieving full remission (Asnis et al. 2004; Berger et al. 2009).

Ketamine, an N-methyl-D-aspartate-type (NMDA) receptor antagonist, has been investigated as a treatment for both treatment-resistant depression (TRD) and PTSD with promising results (Nikolin et al. 2023; Ragnhildstveit et al. 2023). To date, investigations on ketamine for PTSD have primarily used intravenous (IV) infusion. Recently, our group completed an open-label clinical trial using low dose oral ketamine for PTSD symptom reduction (one dose weekly for six weeks in a titrating up manner), with a treatment response rate of 73% one-week post-treatment and 59% one-month post-treatment (Quigley et al. 2025a). The response rate to oral ketamine was comparable to those reported in IV ketamine PTSD trials (Ragnhildstveit et al. 2023). However, oral ketamine offers the advantages of being more practical for use in conventional clinical psychiatry settings and significantly more cost-effective than other administration methods (Andrade 2019; Can et al. 2021; Muhorakeye and Biracyaza 2021).

However, a significant gap in our understanding of ketamine as a treatment for PTSD lies in identifying the biological changes associated with its effects. The investigation of biomarkers associated with ketamine treatment in humans has been notably limited in the literature, even in the more extensively studied field of ketamine for TRD (Berman et al. 2000). Recent reviews summarizing blood biomarkers related to ketamine and TRD identify only eight studies on to the topic, primarily focusing on brain-derived neurotrophic factor (BDNF), serotonin, interleukin (IL)-6, S100β and various metabolomic biomarkers (Johnston et al. 2024; Kumar et al. 2024). Given the 25-year history of ketamine research in TRD, this scarcity is remarkable. Research into ketamine’s potential for PTSD treatment began 20 years ago (Ragnhildstveit et al. 2023), yet the only blood biomarker reported in this area specifically is d-serine, with higher plasma levels linked to shorter remission times in a ketamine-psychotherapy trial (Pradhan et al. 2018). The remainder of the PTSD biomarker literature focuses on biomarker differences between cohorts with and without PTSD or biomarkers that confer risk of developing PTSD (Michopoulos et al. 2015).

Important biomarkers that have been investigated in relation to PTSD include circulating BDNF, where meta-analyses show an overall *increase* in BDNF during the clinical condition of PTSD, opposite to every other psychiatric disorder investigated (Zou et al. 2024). Additionally, immune cytokines often present as a systemic, low-grade inflammation in PTSD patients (Baker et al. 2012; Hori and Kim 2019). Other potential PTSD biomarkers with less extensive literature include serotonin (Oglodek 2022), where PTSD cohorts have been reported as having lower circulating levels and FK506 binding protein 51 (FKBP51) (Li et al. 2020), an important stress response co-chaperone with genetic polymorphisms linked to PTSD development (Mehta et al. 2011). Finally, given the connection between BDNF and vascular endothelial growth factor A (VEGF-A) in antidepressant treatment response (Deyama et al. 2019; Kao et al. 2018), our group recently determined that a cohort of older adults with PTSD had higher levels of VEGF-A compared to non-PTSD matched controls (Quigley et al. 2025c). Given the lack of specific biological investigation related to treatment, a critical first step in understanding how ketamine treatment may lead to the clinical improvements in PTSD is to determine what is happening to these common biomarkers before and after treatment.

To address this critical gap in the literature on the biological effects of ketamine in PTSD treatment, we analysed circulating levels of BDNF, VEGF-A, serotonin, FKBP51 and a panel of cytokines (IL-1β, IL-2, IL-4, IL-6, IL-12p70, IL-17 A and tumour necrosis factor alpha (TNFα)) before and after low dose oral ketamine treatment. This investigation aimed to identify potential biomarker changes and their relationships to PTSD symptomology and ketamine administration.

## Materials and methods

### Study participants

Samples for this analysis were collected from the Oral Ketamine Trial on PTSD (OKTOP), with the full details and primary outcomes of the open-label clinical trial described in (Quigley et al. 2025a). Baseline biomarker data only has been previously reported in the context of other PTSD-related analyses in (Quigley et al. 2025b, c). The trial was approved by the Metro North Health Human Research Ethics Committee (HREC) (Project ID: 42836; HREC/18/QPCH/28), the University of the Sunshine Coast HREC (A181190) and prospectively registered with the Clinical Trials Registry of Australia and New Zealand (ANZCTR trial registration number: ACTRN12618001965291, registered 05 December 2018). Of note, study participants were allowed to remain on previously prescribed psychotropic medications during the trial, with ketamine treatment acting an augmentative treatment. To maximize the number of samples for analysis, trial participants diagnosed with PTSD via the Clinician Administered PTSD Scale for DSM-5 (CAPS-5) were included in this study if blood samples were available from the baseline assessment before ketamine treatment (“pre-treatment”) and at least one sample after ketamine treatment (“post-treatment” - ~2 h after the sixth/final ketamine dose, “follow-up 1-week” – 1–7 days after the sixth/final ketamine dose and/or “follow-up 1-month” – 28–32 days after the sixth/final ketamine dose). This generated a study cohort of *n* = 25 participants (Table 1), consisting of *n* = 25 pre-treatment samples, *n* = 19 post-treatment samples, *n* = 24 follow-up 1-week samples and *n* = 23 follow-up 1-month samples. Additionally, *n* = 14 mid-treatment samples (~ 2 h after the third ketamine dose) were included in the biomarker mean comparison and change analysis. Response to ketamine treatment was defined in the trial as ≥ 50% reduction in PTSD Checklist for DSM-5 (PCL-5) score from pre-treatment (Quigley et al. 2025a), with these samples representing response rates of # responders/# non-responders/# missing samples or outcomes of 15/3/7 at post-treatment, 15/8/2 at follow-up 1-week and 12/11/2 at follow-up 1-month for all 25 participants, respectively.

**Table 1:** Participant demographics

Characteristic	Value*
# Participants	*n* = 25
Sex	16 F / 9 M
Age	47.8 ± 13.0 (22–77)
PTSD duration (years)	18.8 ± 16.2 (2–55)
CAPS-5 score	54.2 ± 7.0 (41–67)
Index trauma	
Childhood trauma	11 (44%)
Military/war	4 (16%)
Sexual assault/abuse and/or DV	4 (16%)
Physical assault/abuse/accident	4 (16%)
Emergency services (work)	1 (4%)
Bereavement (death of child)	1 (4%)
Highest education	
Secondary year 10	1 (4%)
Secondary year 12	4 (16%)
Tertiary	12 (48%)
Post-graduate	6 (24%)
Unknown	2 (8%)
Major Depressive Disorder (MDD)	19 (76%)
Complex PTSD (cPTSD)	3 (12%)
Psychotropic medications	
Benzodiazepines	13 (52%)
SSRIs	4 (16%)
SNRIs	6 (24%)
Tetracyclic antidepressants	3 (12%)
Others	9 (40%)

* Mean ± standard deviation (range) or count (percentage), as appropriate. *CAPS-5*  Clinician-Administered PTSD Scale for DSM-5, *DV*  domestic violence, *SSRI*  Selective serotonin reuptake inhibitors, *SNRI*  Serotonin and norepinephrine reuptake inhibitors

## Clinical measures

In addition to the CAPS-5 (Weathers et al. 2018) conducted at pre-treatment to confirm study eligibility, the following clinical rating scales were completed by participants at pre-treatment, post-treatment and follow-up timepoints to assess study outcomes: (a) PCL-5, a 20-item self-report measure often used in conjunction with the CAPS-5 to monitor PTSD symptom change across patient treatment (Blevins et al. 2015; Weathers et al. 2018); (b) Depression, Anxiety, and Stress Scale (DASS-21), 21-item self-report measure assessing perceived depressive, anxiety, and stress symptoms (Lovibond and Lovibond 1995); and (c) World Health Organization Well-Being Index (WHO-5), a 5-item self-report global rating scale measuring subjective well-being (Bech et al. 2003).

## Sample collection and processing

Non-fasting whole blood was collected at pre-treatment (week 0), mid-treatment (week 3), post-treatment (week 6), follow-up 1-week (week 7) and follow-up 1-month (week 10) timepoints at midday/early afternoon by a certified phlebotomist using serum separator tubes (SST) and K2EDTA (plasma) collection tubes. Samples were held at room temp for 30 min (to allow for serum clotting), before being centrifuged at 2465 x g for 15 min at 4 °C to generate serum and plasma, which was aliquoted and stored at -80 °C. If samples could not be processed immediately after clotting, tubes were transferred to 4 °C for a maximum of 3.5 h before processing continued.

## Biomarker quantification

Before analysis, stored serum and plasma was thawed on ice and centrifuged at 10,000 x g for 10 min at 4 °C to remove any particulates before testing. BDNF and VEGF-A were quantified from both serum and plasma while serotonin was quantified from serum only and FKBP51 and all cytokines were quantified from plasma only. Standard ProcartaPlex simplex assays (ThermoFisher Scientific, Australia) were combined for the detection of BDNF (lower limit of quantification (LLOQ) of 2.03 pg/ml) and VEGF-A (LLOQ of 4.88 pg/ml) while high-sensitivity assays were used to quantify IL-1β (LLOQ of 0.28 pg/ml), IL-2 (LLOQ of 0.88 pg/ml), IL-4 (LLOQ of 1.29 pg/ml), IL-6 (LLOQ of 1.29 pg/ml), IL-12p70 (LLOQ of 0.77 pg/ml), IL-17 A (LLOQ of 0.30 pg/ml) and TNFα (LLOQ of 0.62 pg/ml). Assays were conducted according to the manufacturer’s instruction, read on a Luminex 200 instrument (ThermoFisher) and processed using the ProcartaPlex Analysis App (ThermoFisher). An overall study linearity of dilution of 0.97 ± 0.02 and percent recovery of 100.8 ± 13.1% of Luminex standards was achieved. Enzyme-linked immunosorbent assays (ELISAs) were used to quantify serotonin (Fast Track kit, Immusmol, France; LLOQ of 15 ng/ml) and FKBP51 (Human kit, Invitrogen, ThermoFisher, Australia; LLOQ of 0.41 ng/ml) according to the manufacturer’s instructions and processed using GainData (https://www.arigobio.com/elisa-analysis). All samples from an individual were tested on the same assay plate to reduce within-subject variability. BDNF, VEGF-A and serotonin levels were within assay detectable ranges for all samples tested while detectable cytokine levels ranged from 95% (IL-1β and IL-12p70), 90% (IL-4 and IL-6), 80% (IL-2) and 72% (IL-17 A and TNFα) of samples tested. FKBP51 levels were within the assay detectable range for 66% of samples. Samples with targets below their assay’s LLOQ were assigned a value of half their LLOQ for statistical analysis.

### Statistical analysis

All analyses were performed using R Statistical Software (v4.3.3) (R Core Team 2024). The Skillings–Mack test (Skillings and Mack 1981) was used to compare biomarker repeated measures across five timepoints in an incomplete block design. This nonparametric ANOVA-type test generalizes the Friedman test and accommodates missing data. Analysis was conducted in R using the Skillings.Mack package (Srisuradetchai 2015). Changes in biomarker levels compared to baseline were assessed by linear mixed model analysis using lme4 package (Bates et al. 2015) and lmerTest package (Kuznetsova et al. 2017) and visualised using ggplot2 (Wickham 2016). Models used change values for each biomarker, which were calculated by subtracting log transformed pre-treatment levels from log transformed subsequent timepoint levels (similar to (Zalta et al. 2024). The cytokine heatmap was generated with heatmap.2 (in gplots) (Warnes et al. 2015) after scaling samples within each target (subtract target mean and divide by the target standard deviation). Comparisons of biomarker levels by immune group were calculated using the Wilcoxon rank sum test (also known as the Mann-Whitney U test), with p-values adjusted for multiple comparisons using Benjamini-Hochberg (BH) false discovery rate method (Benjamini and Hochberg 1995). Spearman rank correlations were calculated using rcorr (in Hmisc) (Harrell Jr 2025), visualised using corrplot (Wei 2024) and p-values adjusted using the BH false discovery rate method.

## Results

### Biomarker changes throughout ketamine treatment and follow-up

Average quantities for each biomarker tested are reported in Table 2 by timepoint. BDNF (from plasma), VEGF-A (from serum), IL-2, IL-4 and IL-6 were found to have had a significantly change in their mean biomarker quantity at some point throughout the trial (Table 2). However, because of the nature of the Skillings-Mack repeated measure test with missing data test, post-hoc analyses on individual timepoint differences was not possible.

**Table 2:** Mean blood biomarker levels throughout ketamine treatment trial

Marker	Pre-treatment (*n* = 25)	Mid-treatment (*n* = 14)	Post-treatment (*n* = 19)	Follow-up 1 week (*n* = 24)	Follow-up 1 month (*n* = 23)	Skillings-Mack test (T_SM_)
BDNF (serum) (pg/ml)	261.34 ± 347.78	205.12 ± 294.41	170.94 ± 225.61	219.80 ± 285.80	187.21 ± 202.89	χ^2^ (4) = 6.97, *P* = 0.140
BDNF (plasma) (pg/ml)	8.65 ± 9.08	5.92 ± 8.24	4.01 ± 3.36	7.52 ± 7.66	6.44 ± 7.83	**χ**^**2**^ **(4) = 28.54**,***P*** ** < 0.001**
VEGF-A (serum) (pg/ml)	323.62 ± 269.86	287.21 ± 272.13	309.45 ± 225.03	295.71 ± 262.27	302.61 ± 303.85	**χ**^**2**^ **(4) = 10.37**,***P*** ** = 0.030**
VEGF-A (plasma) (pg/ml)	49.94 ± 48.50	43.47 ± 29.55	42.68 ± 39.35	45.63 ± 33.87	38.06 ± 23.41	χ^2^ (4) = 2.64, *P* = 0.627
Serotonin (ng/ml)	89.95 ± 75.30	ND	105.11 ± 106.96	83.82 ± 83.17	93.72 ± 80.78	χ^2^ (3) = 1.88, *P* = 0.606
FKBP51 (ng/ml)*	20.92 ± 59.62	ND	10.60 ± 28.25	16.99 ± 46.45	17.55 ± 46.75	χ^2^ (3) = 1.34, *P* = 0.585
IL-1β (pg/ml)*	1.88 ± 1.34	2.24 ± 1.89	2.27 ± 1.91	2.91 ± 2.42	2.70 ± 1.82	χ^2^ (4) = 8.10, *P* = 0.082
IL-2 (pg/ml)*	2.24 ± 3.03	3.69 ± 4.42	3.17 ± 4.12	4.01 ± 3.63	3.99 ± 3.51	**χ**^**2**^ **(4) = 12.18**,***P* = 0.007**
IL-4 (pg/ml)*	1.91 ± 1.14	2.81 ± 2.39	2.07 ± 1.43	3.16 ± 2.53	2.95 ± 1.79	**χ**^**2**^ **(4) = 13.18**,***P*** ** = 0.007**
IL-6 (pg/ml)*	4.01 ± 6.47	6.14 ± 11.02	4.62 ± 8.59	4.42 ± 7.03	5.81 ± 11.81	**χ**^**2**^**(4) = 9.53**, ***P ****= ***0.041**
IL-12p70 (pg/ml)*	6.49 ± 4.83	6.30 ± 5.63	4.63 ± 3.31	6.46 ± 5.97	5.94 ± 3.70	χ^2^ (4) = 6.71, *P* = 0.144
IL-17 A (pg/ml)*	0.79 ± 0.66	0.88 ± 0.97	0.61 ± 0.66	1.00 ± 1.12	0.88 ± 0.69	χ^2^ (4) = 7.24, *P* = 0.063
TNFα (pg/ml)*	3.19 ± 4.23	2.92 ± 2.76	2.72 ± 2.53	3.11 ± 2.93	2.70 ± 2.39	χ^2^ (4) = 1.51, *P* = 0.763

Values given as mean ± standard deviation. *ND* Not determined. Asterisks denote targets where calculations include inferred quantities based on assay detection limits (details in Methods). A breakdown of these averages by trauma type (childhood trauma verses other) is given in Online Resource 1

Despite some biomarkers showing significant group-levels changes at some point throughout the oral ketamine trial, significant variation was observed within all biomarkers, as evident by the large standard deviations in Table 2. To address this individual variability and investigate changes further, additional data transformation and analysis was performed with the biomarkers BDNF, VEGF-A, and serotonin (where quantifiable results were obtained from all the samples tested (see methods)). Each participant’s BDNF, VEGF-A and serotonin quantities were log-transformed and subtracted from their pre-treatment values, thereby setting each participant’s pre-treatment value to “zero” and converting subsequent timepoint values to the change in biomarker level from pre-treatment. Linear mixed model analysis was then used to assess the pattern of change. Models considered change in biomarker quantity and study timepoints as fixed effects and individual participants as random effects. The age and sex of the study participants were tested as additional fixed effects and found not to be significant for these biomarkers in this dataset, so were omitted from the final models.

**Fig. 1:**
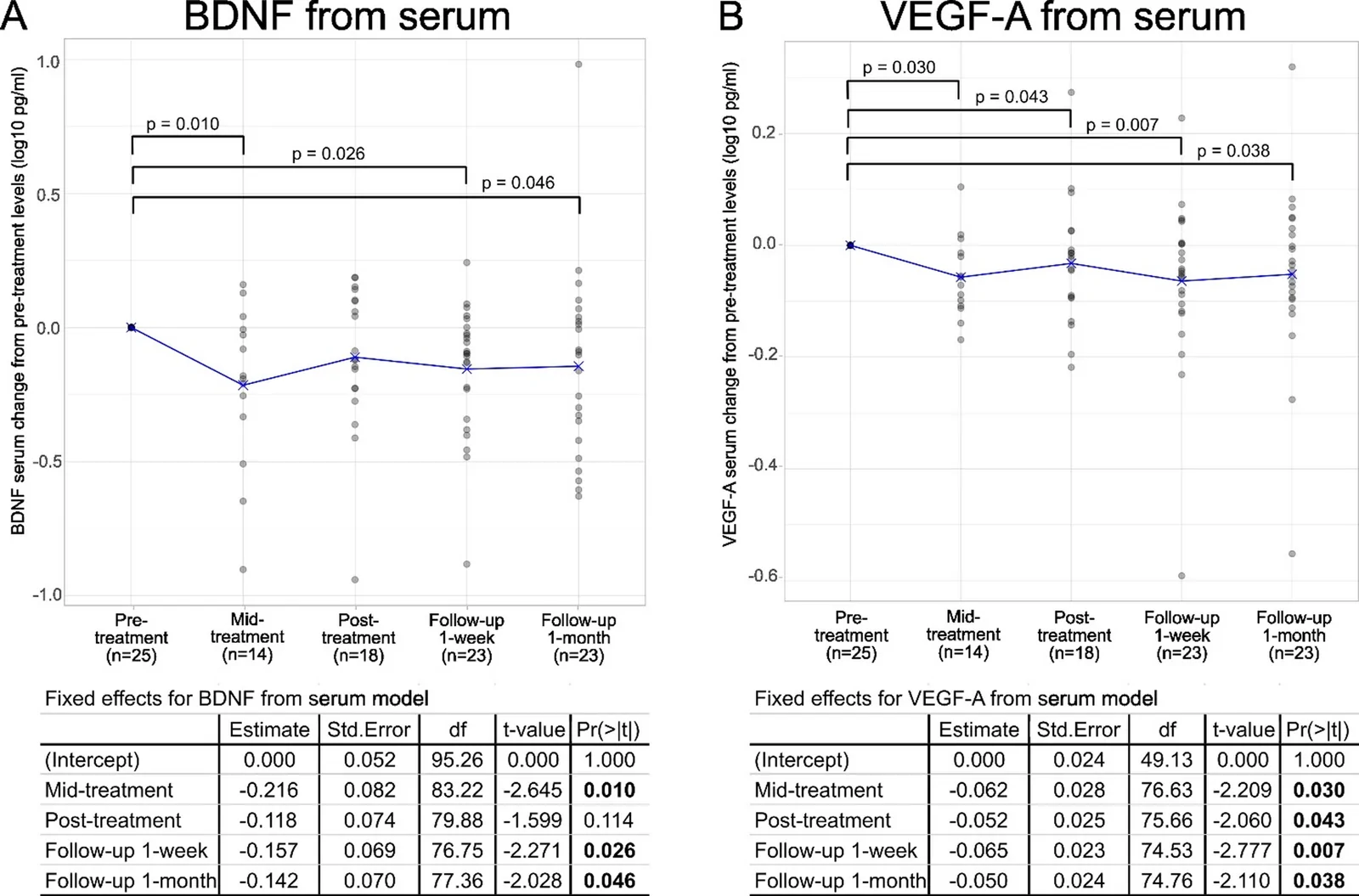
Linear mixed models (LMMs) for BDNF (**A**) and VEGF-A (**B**) levels from serum throughout the trial. Change values for each biomarker were calculated by subtracting log10 pre-treatment values from each log10 timepoint value for each participant. The average change at each timepoint is connected by the blue line and significance differences (*p* < 0.05) compared to pre-treatment are indicated. The fixed effect statistics for each model are given as a table under their respective graphs and full model outputs can be found in Online Resource 2

From this change analysis, BDNF and VEGF-A levels from serum were found to show small but significant decreases after ketamine treatment (Fig. 1). Small BDNF decreases from pre-treatment were detected at mid-treatment (decrease of 0.216 log10 pg/ml, *p* = 0.010), follow-up 1-week (decrease of 0.157 log10 pg/ml, *p* = 0.026) and follow-up 1-month (decrease of 0.142 log10 pg/ml, *p* = 0.046) (Fig. 1A). Concurrently, significant decreases in VEGF-A levels from pre-treatment were detected at mid-treatment (decrease of 0.062 log10 pg/ml, *p* = 0.030), post-treatment (decrease of 0.052 log10 pg/ml, *p* = 0.043), follow-up 1-week (decrease of 0.065 log10 pg/ml, *p* = 0.007) and follow-up 1-month (decrease of 0.050 log10 pg/ml, *p* = 0.038) (Fig. 1B). BDNF, VEGF-A and serotonin levels from plasma did not show any significant change over the course of ketamine treatment (Online Resource 2). Given the lack of significant change in BDNF and VEGF-A from plasma in this more detailed analysis, the remaining analyses in this study focused on BDNF and VEGF-A levels from serum only. Additionally, there was no significant difference in any biomarker pattern throughout the trial by trauma type (childhood verses other trauma) (Online Resource 1).

## Biomarker relationships by inflammatory status of participants

**Fig. 2:**
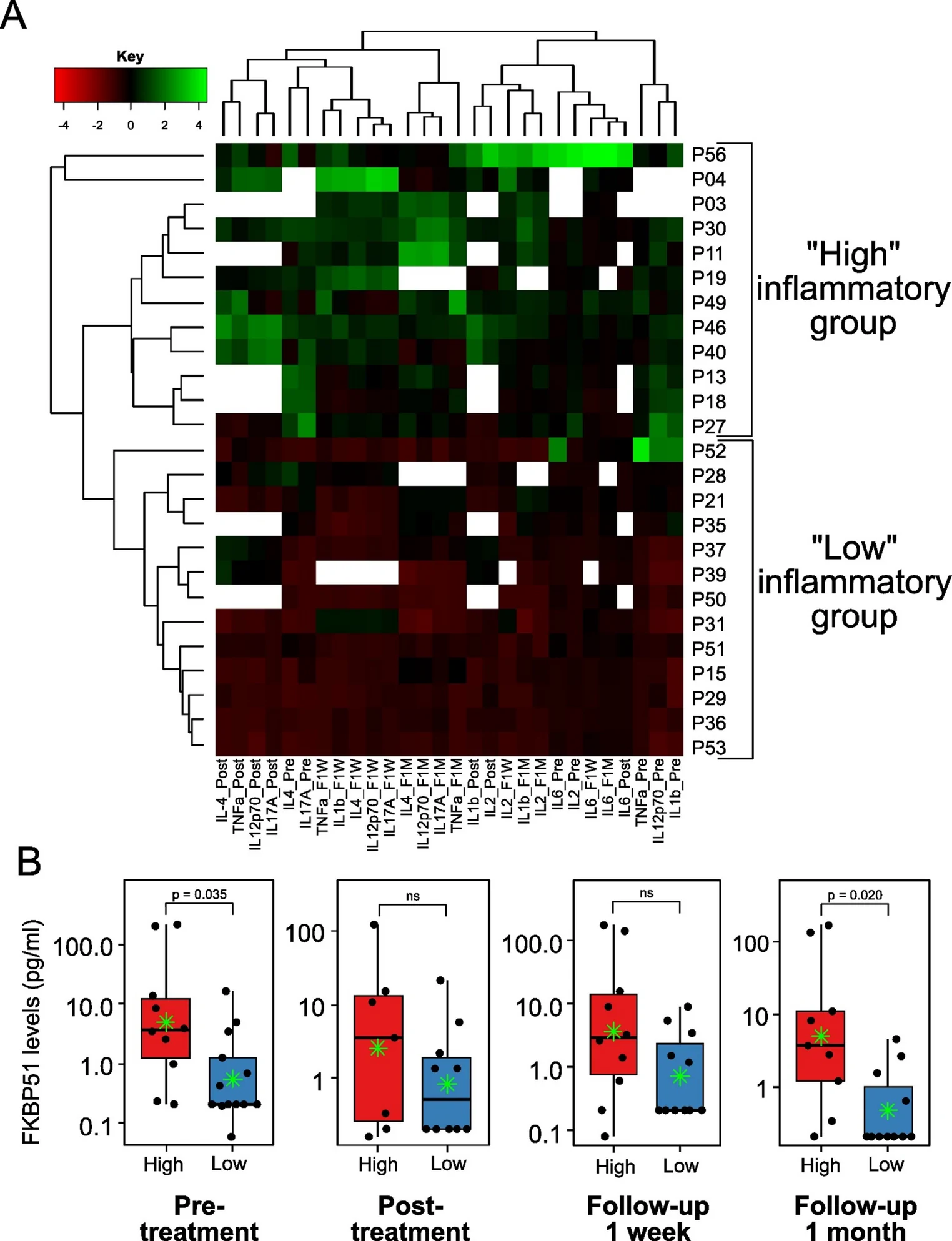
(**A**) Clustering of cytokine levels by participant and study timepoint. P# indicate individual participants (rows). Values for each cytokine at each timepoint (columns) were normalised for comparison (subtract the group mean and divide by the group standard deviation – generates black for average values, green for values above the average and red for values below the average). White squares indicate missing samples. _Pre = pre-treatment sample, _Post = post-treatment sample, _F1W = follow-up 1 week sample, _F1M = follow-up 1 month sample. A breakdown of the inflammatory clusters by trauma type (childhood trauma vs. other trauma types) is presented in Online Resource 1. (**B**) Box and whisker plots of FKBP51 values by inflammatory group status at study timepoints (note: mid-treatment samples were not tested for FKBP51, see Table 2). Green star denotes group mean. Wilcoxon rank sum test was used to determine statistical differences, with p-values adjusted for multiple comparisons using BH false discovery rate. ns = not significant (*p* > 0.05)

To understand the immune status of the study group better and investigate cytokine measurements in more detail, immune biomarkers were normalised and clustered for all study participants and timepoints (Fig. 2A). This clustering revealed two distinct inflammatory groups within the study cohort: a “high” inflammatory group (*n* = 12), with average cytokine levels above the group average (green colours in Fig. 2A) and a “low” inflammatory group (*n* = 13), with average cytokine levels below the group average (red colours in Fig. 2A). Between these inflammatory groups, there was a statistically significant difference between IL-1β, IL-2, IL-4, IL-12p70, IL-17 A and TNFα levels at all four major study timepoints (pre-treatment, post-treatment, follow-up 1-week and follow-up 1-month), with IL-6 having significant differences at pre-treatment and follow-up 1-week (Online Resource 3).

Examination of the inflammatory clusters revealed two important findings. The first was that ketamine did not appear to significantly alter circulating cytokine levels during or after treatment. The levels of each cytokine remained relatively consistent between timepoints (Online Resource 3) and the distinct separation between the inflammatory groups was maintained throughout the treatment course (Fig. 2A). There was no significant correlation or difference detected between a participant’s inflammatory group status and their personal demographics (sex, age, PTSD severity, presence of co-morbid depression or perceived stress at the time of blood collection) or their treatment response in the trial, leaving the reason for the two distinct inflammatory subgroups unknown.

The results for FKBP51, on the other hand, revealed a significant association between FKBP51 levels and inflammatory group status (Fig. 2B). At the four study timepoints where FKBP51 was tested, average FKBP51 levels were consistently higher in the “high” inflammatory group, with this difference reaching statistical significance at the pre-treatment and follow-up 1-month timepoints (Fig. 2B, Online Resource 3). No difference by inflammatory group was detected for BDNF, VEGF-A or serotonin levels from serum for any timepoint in the study (Online Resource 3).

### Correlations between and within blood biomarkers and clinical scales

**Fig. 3:**
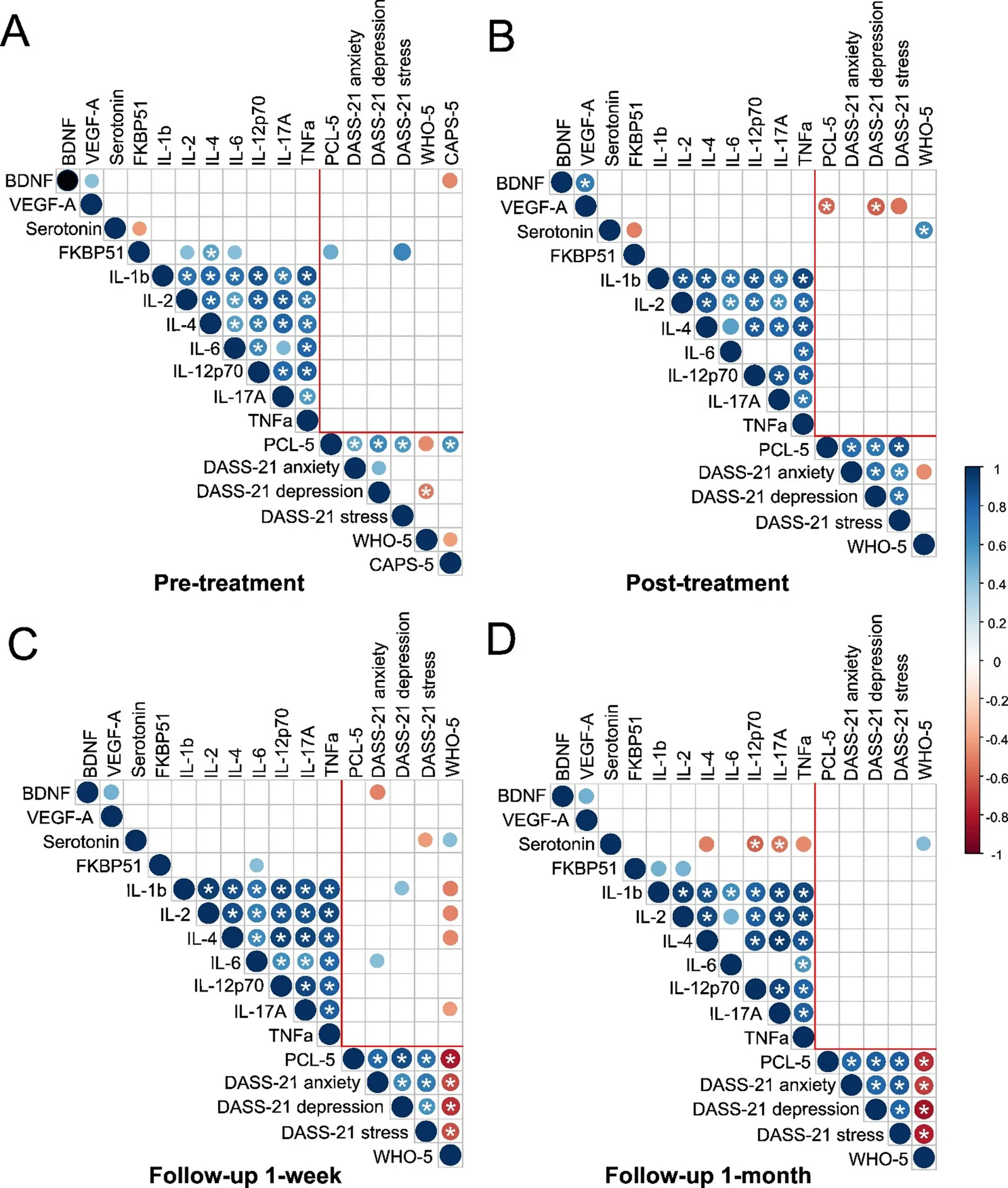
Spearman rank correlations between blood biomarkers and clinical scales at (**A**) pre-treatment, (**B**) post-treatment, (**C)** follow-up 1 week and (**D**) follow-up 1 month. Correlation coefficients are represented by the size and colour (key on right) of the circles. Only statistically significant correlations (*p* < 0.05) are shown. Correlations that remained significant after Benjamini-Hochberg (BH) false discovery rate adjustment for multiple comparisons contain asterisks.​​ BDNF = Brain-derived neurotrophic factor from serum, VEGF-A = Vascular endothelial growth factor A from serum, FKBP51 = FK506-binding protein 51, PCL-5 = PTSD Checklist for DSM-5, DASS-21 = Depression Anxiety Stress Scales, WHO-5 = World Health Organisation Five Well-Being Index, CAPS-5 = Clinician-Administered PTSD Scale for DSM-5. Detailed correlation statistics are presented in Online Resource 4 and a breakdown of correlations by trauma type (childhood trauma vs. other trauma types) is presented in Online Resource 1

At each of the four major study timepoints, correlations were examined between blood biomarkers and study clinical scales (PCL-5, DASS-21 and WHO-5) to identify potential temporal relationships between these measures (Fig. 3, Online Resource 4).

Notably, strong relationships were observed among immune cytokines (IL-1β, IL-2, IL-4, IL-6, IL-12p70, IL-17 A and TNFα) and clinical scales (PCL-5, DASS-21 subscales and WHO-5) across study timepoints (Fig. 3). Immune biomarker comparisons revealed only IL-6 diverged from the overall pattern of strong positive correlation between the cytokines at all the study timepoints. In terms of clinical scales, PCL-5 scores strongly correlated with the other scales at all the timepoints (including CAPS-5 scores at pre-treatment (r_s_(23) = 0.593, *p* = 0.002, p-adjusted = 0.026)). DASS-21 subscales for anxiety, depression and stress and WHO-5 showed strengthening correlation as participants completed the ketamine treatment and continued to follow-up assessments (Fig. 3).

Examining the non-immune blood biomarkers, BDNF and VEGF-A levels from serum appeared to only correlate to each other, with positive correlations detected at all timepoints throughout the study (Fig. 3; pre-treatment: r_s_(23) = 0.415, *p* = 0.039, p-adjusted = 0.149; post-treatment: r_s_(17) = 0.691, *p* = 0.001, p-adjusted = 0.006; follow-up 1-week: r_s_(22) = 0.478, *p* = 0.018, p-adjusted = 0.054; follow-up 1-month: r_s_(21) = 0.472, *p* = 0.023, p-adjusted = 0.075)). Serotonin levels were negatively correlated with FKBP51 at pre- and post-treatment (pre-treatment: r_s_(23) = 0.415, *p* = 0.039, p-adjusted = 0.149; post-treatment: r_s_(17) = 0.691, *p* = 0.001, p-adjusted = 0.006) and a subset of cytokines (IL-4, IL-12p70, IL-17 A and TNFα) at follow-up 1-month (Fig. 3). Finally, in addition to a relationship with serotonin, FKBP51 also exhibited sporadic correlations with cytokines IL-1β, IL-2, IL-4 and IL-6 at various timepoints across the trial (Fig. 3, Online Resource 4).

Examining the relationships between blood biomarkers and clinical scales, several noteworthy correlations emerged. In relation to PTSD symptoms, only BDNF levels from serum correlated negatively with CAPS-5 scores at pre-treatment (r_s_(23)=-0.487, *p* = 0.014, p-adjusted = 0.080) (Fig. 3A), while FKBP51 levels correlated positively with PCL-5 at pre-treatment (r_s_(21) = 0.497, *p* = 0.016, p-adjusted = 0.072) and VEGF-A levels correlated negatively with PCL-5 at post-treatment (r_s_(16)=-0.509, *p* = 0.011, p-adjusted = 0.039) (Fig. 3A and B, respectively). Next, serotonin levels were consistently positively correlated with WHO-5 scores after ketamine treatment (post-treatment: r_s_(16) = 0.632, *p* = 0.005, p-adjusted = 0.022; follow-up 1-week: r_s_(20) = 0.425, *p* = 0.48, p-adjusted = 0.111; follow-up 1-month: r_s_(19) = 0.440, *p* = 0.046, p-adjusted = 0.138) (Fig. 3, Online Resource 4).

## Discussion

The present study represents the first broad analysis of blood biomarkers to explore biological changes and relationships associated with ketamine treatment for PTSD. By investigating these mechanisms, our work takes an important step towards addressing the significant gap in understanding of how ketamine facilitates PTSD symptom improvement. We have identified key, albeit small, changes in BDNF and VEGF-A levels following treatment, along with significant associations between blood biomarkers and PTSD clinical measures.

BDNF has been extensively investigated across a range of mental health conditions, including PTSD. It assumes a crucial role in neuronal survival, growth and plasticity and, as such, is essential for learning and memory development (Bathina and Das 2015). Previous studies by our team (as well as many others) have reported relationships between circulating BDNF and PTSD (Quigley et al. 2025b; Zalta et al. 2024). These relationships include correlations between BDNF levels and PTSD symptom severity (Quigley et al. 2025b; Zalta et al. 2024), as well as differences in BDNF levels between cohorts with and without PTSD (Quigley et al. 2025c; Zou et al. 2024). Interestingly, while meta-analysis of the literature has found BDNF levels *lower* in cohorts with major depressive disorder (MDD), bipolar disorder, schizophrenia, panic disorder or obsessive-compulsive disorder, the same meta-analysis reported that BDNF levels are *higher* in cohorts with PTSD (Zou et al. 2024). This observation suggests that PTSD may have distinct biological characteristics compared to the other psychiatric conditions, even though depression (as characterised by MDD) is often co-occurring. Moreover, it may be that BDNF might be considered a “goldilocks protein”, where levels that are either too low or too high contribute to the emergence of symptoms. Since elevated BDNF levels are often observed in PTSD, the small but significant decrease detected in our cohort from serum following ketamine treatment (Fig. 2A) may suggests a potential biological mechanism through which ketamine contributes to PTSD symptom improvement. This supports the notion that BDNF increases in reaction to stress-induced neuronal damage but decreases when the stressor (i.e., PTSD symptoms) is mitigated (Guo et al. 2019).

Interestingly, reducing elevated levels of BDNF may be a biomarker of general PTSD symptom improvement and not limited to just ketamine treatment. (Zalta et al. 2024) recently reported the outcome of a 3-week cognitive processing therapy (CPT) treatment for military veterans with PTSD, showing that while BDNF levels at post-treatment study timepoints negatively correlated with PTSD symptoms, the change in each individual’s BDNF level from pre-treatment to post-treatment showed an overall significant decrease, similar to the findings in this study (Fig. 2A). Similarly to our present study (Table 1), (Zalta et al. 2024) also reported a wide variation in pre- and post-treatment BDNF levels among individual. This suggests that there may not be a universal “optimal” BDNF level, but rather that each individual has a unique baseline influenced by genetic and/or biological factors. Disruptions to this balance may only become apparent though broad comparisons between PTSD and control cohorts (as seen in previous literature (Quigley et al. 2025c; Zou et al. 2024) or through longitudinal individual BDNF analysis (as observed in this study and (Zalta et al. 2024).

The relationship between BDNF and PTSD also involves more dimensions than just a general amount in circulation. Our previous work has determined that the form of BDNF being measured (proBDNF verses mature BDNF verses total BDNF) is important in detecting correlations with PTSD symptom severity (Quigley et al. 2025b), and that serum but not plasma levels differentiated PTSD from non-PTSD cohorts (Quigley et al. 2025c). The difference between plasma BDNF levels (representing only freely circulating BDNF) and serum BDNF levels (which reflect both circulating plasma levels and platelet-stored BDNF reserves) are significant and have led to the suggestion that plasma BDNF levels may reflect cerebral levels more closely while serum BDNF associations suggest that platelet biology may have an underappreciated role in mental health (Bouhaddou et al. 2024). Finally, it is important to acknowledge that BDNF in all its forms has important links to many mental health and neurological conditions, and while it is unlikely that any BDNF measurement alone will be distinct enough to serve as an independent biomarker for PTSD, investigations into the complex relationship between BDNF and PTSD will continue to advance our understanding of how this important neurotrophin is involved in mental health.

The findings from the present study show a correlation between VEGF-A and BDNF serum levels (Fig. 3A-D) and a parallel VEGF-A serum level decrease after ketamine treatment (Fig. 1B), supporting existing evidence that highlight the interdependence of BDNF and VEGF-A in mediating ketamine’s therapeutic effects. VEGF-A is best known for its roles in angiogenesis and blood vessel permeability but, like BDNF, it also has recognised neurotropic activity (Sondell et al. 1999). Both BDNF and VEGF-A have been linked genetically to antidepressant treatment responses in people (Kao et al. 2018) and rodent studies have identified that the sustained antidepressant action of ketamine requires a reciprocal interdependence of both BDNF and VEGF-A on each other and the brain (Deyama et al. 2019). Rodent models of depression have found that ketamine rapidly increases BDNF and VEGF-A levels in the medial prefrontal cortex, which increases the number and function of synaptic spines, and in the hippocampus, which enhances neurogenesis and contributes to the antidepressant effects observed (Deyama and Duman 2020). How these processes differ in PTSD (where our previous work has also detected elevated levels of VEGF-A in a PTSD cohort verses controls (Quigley et al. 2025c)) remains to be elucidated. Interestingly, at the post-treatment timepoint in this study, BDNF from serum did not significantly correlate with any clinical scales, but VEGF-A levels from serum significantly negatively correlated with PCL-5 and DASS depression and stress subscales and significantly positively correlated with BDNF at the same time (Fig. 3B). Although small study sample sizes and individual variability in biomarkers can influence the detection of statistically significant relationships, the consistent associations observed between BDNF, VEGF-A and the clinical scales before and after treatment reinforce the notion that a biologically meaningful mechanism may be at play and that the present findings warrant further investigation.

The other group of significant biomarkers commonly investigated in relation to mental health (and PTSD) are the immune cytokines. A dysregulation of the immune system, commonly reported as a systemic, low-grade inflammation, has been observed in individuals with PTSD (Baker et al. 2012; Hori and Kim 2019). In the present study, half of our trial participants appeared to have low-level inflammation, while the other half did not (Fig. 2). The reason for this subgrouping was not apparent from the demographic information collected and did not appear to impact treatment response with ketamine. However, the variability in immune status within our PTSD cohort may help explain why other studies do not always detect significant immune differences related to PTSD status (Passos et al. 2015; Renna et al. 2018; Yang and Jiang 2020). In the present study, there did not appear to be a consistent detectable change in these circulating cytokine levels throughout the treatment and follow-up, but additional investigations with larger cohort sizes and broader, more sensitive panels are needed before any definitive statements can be made about immune biomarkers and changes related to ketamine treatment in PTSD.

Finally, despite their recognised roles in wellbeing and stress responses, research on serotonin and FKBP51 in the context of PTSD remains limited. Serotonin dysfunction has been linked to the pathophysiology of trauma-related symptoms associated with PTSD (Davis et al. 1997), while abnormal increases in FKBP51 complexed with its glucocorticoid receptor appear to lead to glucocorticoid resistance, hyperarousal of the stress-response system, and symptoms of PTSD (Li et al. 2020). Previous research in mice has detected increased gene expression of FKBP51 in serotonergic neurons from the dorsal raphe after stress (Lesiak et al. 2021), while human genotype-phenotype analysis has found FKBP51 single nucleotide polymorphisms influenced SSRI treatment outcomes in MDD (Ellsworth et al. 2013). Work by (Klengel et al. 2013) has also shown that childhood trauma (which represents almost half of the index PTSD trauma type for participants in this study) was associated with DNA methylation in specific *FKBP5* functional glucocorticoid response elements, leading to global gene expression changes in the immune system. These genetic childhood-induced trauma changes within *FKBP5* resulted in higher risks of trauma-associated psychiatric, immune and metabolic disorders in exposed adults (Klengel et al. 2013), which may help explain the association between the inflammatory groups and FKBP51 levels detected in this study (Fig. 2).

To our knowledge, this is the first study to report a significant relationship between circulating serotonin and FKBP51 levels, finding these biomarkers were negatively correlated during PTSD and immediately after treatment (Fig. 3A and B). Notably, the correlation did not persist at the follow-up timepoints, prompting questions about how PTSD symptomology and its improvement may impact this relationship. Related to this was the fact that serotonin levels positively correlated with WHO-5 wellbeing scores, but only after ketamine treatment (Fig. 3B, C and D). This corroborates other research that has reported whole blood serotonin levels can relate to wellbeing (de Vries et al. 2022), but that relationship was only seen as positively related to positive affect but not related to negative affect (Williams et al. 2006). As such, the serotonin-wellbeing relationship was only detected once participants started feeling better. Alternatively, FKBP51 levels positively correlate with PCL-5 and DASS-21 stress scores at pre-treatment, suggesting its levels relate more strongly to the stress response linked to active PTSD.

There were limitations to this study that need to be acknowledged and considered when interpreting the results. The small sample size and incomplete sample sets for some participants limit the generalisability of the study findings. This should be considered, especially when certain changes or relationships were not detected (i.e. cytokine results and differences by trauma type in Online Resource 1). When relationships were detected, the small study numbers in combination with multiple comparisons caused several interesting correlations to lose statistical significance after p-value correction. To give the reader as much objective information as possible, both uncorrected and corrected p-vales were reported and relationships with supporting evidence in the literature were discussed. However, all the relationships detected in this study need replication in independent, larger cohorts. The samples for this analysis were also derived from an open-label (non-randomised, unblinded) clinical trial using oral ketamine for PTSD treatment and this may have introduced bias in the self-reported clinical scales. Furthermore, study participants were allowed to continue their existing medications during the ketamine treatment trial, thus the possibility of combined or synergic effects of ketamine with other medications cannot be ruled out. Despite these limitations, many statistically significant changes and relationships were still detectable within the study cohort, even after correcting for multiple comparisons, and findings were generally consistent or logical in the context of published literature.

In conclusion, this study reports the first broad analysis of blood biomarker in participants with PTSD before and after ketamine treatment. Significant decreases in BDNF and VEGF-A levels from serum were detected following treatment with ketamine, consistent with established knowledge that BDNF is typically elevated during PTSD and suggesting that BNDF may be a “goldilocks protein” that requires levels to be “just right” for the optimal mental health of individuals. This study adds new and significant information to our understanding of serotonin and FKBP51 in PTSD and symptom improvement. Finally, this study highlights the significant interpersonal variability in blood biomarker levels and reinforces the power of individual participant longitudinal analysis to understand important biological changes during illness and treatment.

## Supplementary Information

Below is the link to the electronic supplementary material.

Supplementary Material 1

Supplementary Material 2

Supplementary Material 3

Supplementary Material 4

Supplementary Material 5

## Data Availability

The datasets for this study can be available from the corresponding author upon reasonable request.
