# Supplementary material for: Blood biomarker changes and relationships after low dose oral ketamine treatment for post-traumatic stress disorder (PTSD)
Source: Psychopharmacology (Berl). 2026 Mar 16;243(11):2635–47. doi: 10.1007/s00213-026-07043-6 (PMC13710620; doi:10.1007/s00213-026-07043-6)
Supplement: Supplementary file 1 [file 213_2026_7043_MOESM1_ESM.docx]

February 25, 2026

Psychopharmacology

To Dr Amy Janes, Principal Editor,

**RE: Blood biomarker changes after ketamine treatment for PTSD revised manuscript by Quigley et al. for publication consideration**

We are delighted that you have found our manuscript “Blood biomarker changes and relationships after low dose oral ketamine treatment for post-traumatic stress disorder (PTSD)” acceptable for publication after we complete the corrections suggested by the reviewers. We have now addressed these additional comments, and we thank the reviewers for the excellent feedback and ongoing commitment to improving our work.

Please find included with this response a detailed response to the new reviewer comments, our tracked changes manuscript, and a “clean” version of the manuscript with the modifications for your review. If you require any additional edits to meet you publication requirement, please let me know and I’ll see to them right away.

Many thanks again.

Sincerely,

Dr Bonnie Quigley, Thompson Institute, University of the Sunshine Coast
