# Supplementary material for: Blood biomarker changes and relationships after low dose oral ketamine treatment for post-traumatic stress disorder (PTSD)
Source: Psychopharmacology (Berl). 2026 Mar 16;243(11):2635–47. doi: 10.1007/s00213-026-07043-6 (PMC13710620; doi:10.1007/s00213-026-07043-6)
Supplement: Supplementary file 2 [file 213_2026_7043_MOESM2_ESM.docx]

**Online Resource 2**

Complete Linear mixed model output related to Figure 1.

BDNF serum LMM output (Figure 1A)

```
> m1 <- lmer(BDNF_S_LOG ~ Timepoint + (1 | StudyID), data = df1)
> summary(m1)

Linear mixed model fit by REML. t-tests use Satterthwaite's method [
lmerModLmerTest]
Formula: BDNF_S_LOG ~ Timepoint + (1 | StudyID)
   Data: df1

REML criterion at convergence: 25.9

Scaled residuals:
    Min      1Q  Median      3Q     Max
-2.9008 -0.3951  0.0498  0.5019  4.2276

Random effects:
 Groups   Name        Variance Std.Dev.
 StudyID  (Intercept) 0.008185 0.09047
 Residual             0.058451 0.24177
Number of obs: 105, groups:  StudyID, 25

Fixed effects:
              Estimate Std. Error         df t value Pr(>|t|)
(Intercept)  1.782e-16  5.163e-02  9.526e+01   0.000  1.00000
TimepointT3 -2.163e-01  8.176e-02  8.322e+01  -2.645  0.00976 **
TimepointT6 -1.184e-01  7.406e-02  7.988e+01  -1.599  0.11380
TimepointF1 -1.571e-01  6.915e-02  7.675e+01  -2.271  0.02593 *
TimepointF2 -1.419e-01  6.999e-02  7.736e+01  -2.028  0.04601 *
---
Signif. codes:  0 ‘***’ 0.001 ‘**’ 0.01 ‘*’ 0.05 ‘.’ 0.1 ‘ ’ 1

Correlation of Fixed Effects:
            (Intr) TmpnT3 TmpnT6 TmpnF1
TimepointT3 -0.554
TimepointT6 -0.611  0.391
TimepointF1 -0.655  0.412  0.456
TimepointF2 -0.647  0.409  0.450  0.483
-------------------------------------------------------------------
```

VEGF-A serum LMM output (Figure 1B)

```
> m1 <- lmer(VEGF_LOG ~ Timepoint + (1 | StudyID), data = df)
> summary(m1)

Linear mixed model fit by REML. t-tests use Satterthwaite's method [
lmerModLmerTest]
Formula: VEGF_LOG ~ Timepoint + (1 | StudyID)
   Data: df

REML criterion at convergence: -161

Scaled residuals:
    Min      1Q  Median      3Q     Max
-3.1922 -0.4929  0.0406  0.4652  3.3042

Random effects:
 Groups   Name        Variance Std.Dev.
 StudyID  (Intercept) 0.007823 0.08845
 Residual             0.006576 0.08109
Number of obs: 105, groups:  StudyID, 25

Fixed effects:
              Estimate Std. Error         df t value Pr(>|t|)
(Intercept)  9.974e-17  2.400e-02  4.913e+01   0.000  1.00000
TimepointT3 -6.169e-02  2.793e-02  7.663e+01  -2.209  0.03015 *
TimepointT6 -5.168e-02  2.509e-02  7.566e+01  -2.060  0.04282 *
TimepointF1 -6.450e-02  2.322e-02  7.453e+01  -2.777  0.00693 **
TimepointF2 -4.968e-02  2.354e-02  7.476e+01  -2.110  0.03819 *
---
Signif. codes:  0 ‘***’ 0.001 ‘**’ 0.01 ‘*’ 0.05 ‘.’ 0.1 ‘ ’ 1

Correlation of Fixed Effects:
            (Intr) TmpnT3 TmpnT6 TmpnF1
TimepointT3 -0.392
TimepointT6 -0.437  0.388
TimepointF1 -0.472  0.402  0.450
TimepointF2 -0.466  0.401  0.442  0.481
--------------------------------------------------------------------
```

BDNF plasma LMM output (not presented in main paper, as results were not significant)

```
> m1 <- lmer(BDNF_P_LOG ~ Timepoint + (1 | StudyID), data = df1)
> summary(m1)

Linear mixed model fit by REML. t-tests use Satterthwaite's method [
lmerModLmerTest]
Formula: BDNF_P_LOG ~ Timepoint + (1 | StudyID)
   Data: df1

REML criterion at convergence: 88.9

Scaled residuals:
     Min       1Q   Median       3Q      Max
-2.20470 -0.38996  0.05891  0.48217  2.30601

Random effects:
 Groups   Name        Variance Std.Dev.
 StudyID  (Intercept) 0.03467  0.1862
 Residual             0.10587  0.3254
Number of obs: 98, groups:  StudyID, 23

Fixed effects:
              Estimate Std. Error         df t value Pr(>|t|)
(Intercept)  1.742e-17  7.817e-02  7.764e+01   0.000   1.0000
TimepointT3 -2.415e-01  1.123e-01  7.543e+01  -2.151   0.0347 *
TimepointT6 -8.873e-02  1.034e-01  7.364e+01  -0.858   0.3935
TimepointF1 -5.224e-02  9.719e-02  7.181e+01  -0.538   0.5926
TimepointF2 -1.521e-01  9.855e-02  7.227e+01  -1.544   0.1270
---
Signif. codes:  0 ‘***’ 0.001 ‘**’ 0.01 ‘*’ 0.05 ‘.’ 0.1 ‘ ’ 1

Correlation of Fixed Effects:
            (Intr) TmpnT3 TmpnT6 TmpnF1
TimepointT3 -0.524
TimepointT6 -0.570  0.403
TimepointF1 -0.606  0.420  0.457
TimepointF2 -0.598  0.417  0.450  0.480
----------------------------------------------------------------------
```

VEGF-A plasma LMM output (not presented in main paper, as results were not significant)

```
> m1 <- lmer(VEGFA_P_LOG ~ Timepoint + (1 | StudyID), data = df1)
> summary(m1)
Linear mixed model fit by REML. t-tests use Satterthwaite's method [
lmerModLmerTest]
Formula: VEGFA_P_LOG ~ Timepoint + (1 | StudyID)
   Data: df1

REML criterion at convergence: -31.2

Scaled residuals:
    Min      1Q  Median      3Q     Max
-5.0094 -0.2836  0.0726  0.5755  1.4342

Random effects:
 Groups   Name        Variance Std.Dev.
 StudyID  (Intercept) 0.01335  0.1155
 Residual             0.02745  0.1657
Number of obs: 98, groups:  StudyID, 23

Fixed effects:
              Estimate Std. Error         df t value Pr(>|t|)
(Intercept) -7.398e-17  4.212e-02  6.795e+01   0.000    1.000
TimepointT3  5.650e-02  5.736e-02  7.370e+01   0.985    0.328
TimepointT6  5.883e-02  5.272e-02  7.223e+01   1.116    0.268
TimepointF1  1.790e-02  4.950e-02  7.067e+01   0.362    0.719
TimepointF2 -4.728e-02  5.021e-02  7.107e+01  -0.942    0.350

Correlation of Fixed Effects:
            (Intr) TmpnT3 TmpnT6 TmpnF1
TimepointT3 -0.494
TimepointT6 -0.537  0.402
TimepointF1 -0.572  0.418  0.456
TimepointF2 -0.564  0.416  0.448  0.480
------------------------------------------------------------------------
```

Serotonin LMM output (not presented in main paper, as results were not significant)

```
> m1 <- lmer(Serotonin_LOG ~ Timepoint + (1 | StudyID), data = df1)
> summary(m1)

Linear mixed model fit by REML. t-tests use Satterthwaite's method [
lmerModLmerTest]
Formula: Serotonin_LOG ~ Timepoint + (1 | StudyID)
   Data: df1

REML criterion at convergence: -56.8

Scaled residuals:
    Min      1Q  Median      3Q     Max
-4.6130 -0.1100  0.0118  0.3485  3.7526

Random effects:
 Groups   Name        Variance Std.Dev.
 StudyID  (Intercept) 0.001101 0.03318
 Residual             0.025357 0.15924
Number of obs: 91, groups:  StudyID, 25

Fixed effects:
              Estimate Std. Error         df t value Pr(>|t|)
(Intercept) -7.836e-18  3.253e-02  8.665e+01   0.000    1.000
TimepointT6  1.708e-02  4.859e-02  6.979e+01   0.352    0.726
TimepointF1 -4.566e-02  4.552e-02  6.645e+01  -1.003    0.320
TimepointF2 -2.120e-02  4.604e-02  6.707e+01  -0.461    0.647

Correlation of Fixed Effects:
            (Intr) TmpnT6 TmpnF1
TimepointT6 -0.642
TimepointF1 -0.685  0.458
TimepointF2 -0.677  0.453  0.484
----------------------------------------------------------------------
```
