# Supplementary material for: Blood biomarker changes and relationships after low dose oral ketamine treatment for post-traumatic stress disorder (PTSD)
Source: Psychopharmacology (Berl). 2026 Mar 16;243(11):2635–47. doi: 10.1007/s00213-026-07043-6 (PMC13710620; doi:10.1007/s00213-026-07043-6)
Supplement: Supplementary file 3 [file 213_2026_7043_MOESM5_ESM.docx]

**Online Resource 1 - Analyses by trauma type – childhood vs other**

**Table 1 – Results divided by childhood trauma**

**Participants with childhood trauma (n=11)**

| **Marker** | **Pre-treatment** | **Mid-treatment** | **Post-treatment** | **Follow-up 1 week** | **Follow-up 1 month** | **Skillings-Mack test (T_SM_)** |
| --- | --- | --- | --- | --- | --- | --- |
| BDNF (serum) (pg/ml) | 217.35 ± 356.39 | 179.94 ± 352.11 | 170.24 ± 266.41 | 192.90 ± 278.88 | 114.39 ± 93.66 | χ^2^ (4) = 5.12, P = 0.282 |
| BDNF (plasma) (pg/ml) | 8.97 ± 7.48 | 5.09 ± 6.88 | 4.83 ± 4.25 | 8.02 ± 7.25 | 5.01 ± 4.76 | **χ^2^ (4) = 17.62, P < 0.001** |
| VEGF-A (serum) (pg/ml) | 226.32 ± 126.61 | 173.52 ± 93.27 | 210.93 ± 132.78 | 196.46 ± 132.00 | 211.09 ± 124.69 | **χ^2^ (4) = 12.27, P = 0.009** |
| VEGF-A (plasma) (pg/ml) | 32.53 ± 23.31 | 37.63 ± 24.59 | 30.07 ± 19.68 | 32.58 ± 22.31 | 29.44 ± 25.19 | χ^2^ (4) = 1.90, P = 0.765 |
| Serotonin (ng/ml) | 110.79 ± 89.92 | ND | 127.22 ± 136.60 | 105.70 ± 107.27 | 120.86 ± 93.25 | χ^2^ (3) = 2.46, P = 0.497 |
| FKBP51 (ng/ml)* | 24.89 ± 66.33 | ND | 17.31 ± 38.01 | 20.23 ± 47.13 | 20.47 ± 53.90 | χ^2^ (3) = 0.515, P = 0.904 |
| IL-1β (pg/ml)* | 2.03 ± 1.33 | 2.80 ± 2.25 | 2.87 ± 2.09 | 3.77 ± 2.95 | 3.24 ± 2.12 | χ^2^ (4) = 3.93, P = 0.416 |
| IL-2 (pg/ml)* | 3.10 ± 4.42 | 4.80 ± 5.61 | 4.23 ± 5.27 | 5.27 ± 4.87 | 4.81 ± 4.54 | χ^2^ (4) = 6.61, P = 0.099 |
| IL-4 (pg/ml)* | 2.11 ± 1.15 | 3.24 ± 2.86 | 2.47 ± 1.52 | 3.38 ± 3.05 | 2.82 ± 1.49 | χ^2^ (4) = 3.95, P = 0.423 |
| IL-6 (pg/ml)* | 5.37 ± 8.30 | 8.68 ± 14.33 | 6.97 ± 11.52 | 7.47 ± 10.27 | 9.19 ± 16.74 | χ^2^ (4) = 5.76, P = 0.214 |
| IL-12p70 (pg/ml)* | 6.08 ± 5.10 | 6.84 ± 6.75 | 5.18 ± 3.48 | 7.28 ± 8.18 | 5.45 ± 3.43 | χ^2^ (4) = 2.91, P = 0.578 |
| IL-17A (pg/ml)* | 0.81 ± 0.72 | 1.00 ± 1.17 | 0.66 ± 0.73 | 1.16 ± 1.49 | 0.87 ± 0.62 | χ^2^ (4) = 5.36, P = 0.183 |
| TNFα (pg/ml)* | 2.76 ± 2.20 | 3.60 ± 3.24 | 3.50 ± 2.90 | 4.03 ± 3.75 | 3.26 ± 2.91 | χ^2^ (4) = 2.75, P = 0.549 |

**Participants without childhood trauma (n=14)**

| **Marker** | **Pre-treatment** | **Mid-treatment** | **Post-treatment** | **Follow-up 1 week** | **Follow-up 1 month** | **Skillings-Mack test (T_SM_)** |
| --- | --- | --- | --- | --- | --- | --- |
| BDNF (serum) (pg/ml) | 295.91 ± 350.24 | 238.70 ± 222.30 | 171.72 ± 186.20 | 239.02 ± 299.53 | 253.97 ± 253.36 | χ^2^ (4) = 2.96, P = 0.580 |
| BDNF (plasma) (pg/ml) | 8.41 ± 10.45 | 7.01 ± 10.37 | 3.09 ± 1.81 | 7.17 ± 8.19 | 7.75 ± 9.90 | **χ^2^ (4) = 11.64, P = 0.007** |
| VEGF-A (serum) (pg/ml) | 400.06 ± 328.35 | 438.79 ± 363.48 | 418.93 ± 261.71 | 366.61 ± 310.87 | 386.50 ± 393.02 | χ^2^ (4) = 2.48, P = 0.668 |
| VEGF-A (plasma) (pg/ml) | 63.34 ± 58.81 | 51.26 ± 36.00 | 56.69 ± 51.27 | 54.96 ± 38.22 | 45.97 ±19.40 | χ^2^ (4) = 4.86, P = 0.309 |
| Serotonin (ng/ml) | 73.59 ± 59.94 | ND | 80.55 ± 58.82 | 68.20 ± 60.34 | 68.85 ± 61.09 | χ^2^ (3) = 2.04, P = 0.582 |
| FKBP51 (ng/ml)* | 17.87 ± 56.51 | ND | 3.06 ± 7.12 | 14.75 ± 47.77 | 14.89 ± 41.71 | χ^2^ (3) = 1.62, P = 0.431 |
| IL-1β (pg/ml)* | 1.77 ± 1.38 | 1.49 ± 1.02 | 1.61 ± 1.53 | 2.29 ± 1.82 | 2.21 ± 1.42 | χ^2^ (4) = 4.25, P = 0.382 |
| IL-2 (pg/ml)* | 1.58 ± 1.03 | 2.22 ± 1.47 | 1.99 ± 1.99 | 3.11 ± 2.18 | 3.24 ± 2.16 | χ^2^ (4) = 5.76, P = 0.180 |
| IL-4 (pg/ml)* | 1.76 ± 1.15 | 2.24 ± 1.62 | 1.62 ± 1.27 | 3.01 ± 2.20 | 3.07 ± 2.08 | **χ^2^ (4) = 10.49, P = 0.023** |
| IL-6 (pg/ml)* | 2.96 ± 4.72 | 2.75 ± 1.91 | 2.00 ± 1.48 | 2.23 ± 1.48 | 2.71 ± 1.49 | χ^2^ (4) = 6.75, P = 0.134 |
| IL-12p70 (pg/ml)* | 6.80 ± 4.79 | 5.59 ± 4.18 | 4.02 ± 3.19 | 5.88 ± 3.98 | 6.38 ± 4.02 | χ^2^ (4) = 4.89, P = 0.311 |
| IL-17A (pg/ml)* | 0.78 ± 0.65 | 0.71 ± 0.69 | 0.55 ± 0.62 | 0.89 ± 0.80 | 0.88 ± 0.78 | χ^2^ (4) = 3.44, P = 0.376 |
| TNFα (pg/ml)* | 3.52 ± 5.38 | 1.99 ± 1.82 | 1.85 ± 1.84 | 2.45 ± 2.07 | 2.19 ± 1.78 | χ^2^ (4) = 0.11, P = 0.998 |

**Figure 1 – change in biomarkers throughout the trial by trauma type**

BDNF serum by trauma type LMM output

Graph of the BDNF serum change by trauma type (childhood trauma vs other)

```
refitting model(s) with ML (instead of REML)
Data: df6
Models:
m1: BDNF_S_LOG ~ Timepoint + (1 | StudyID)
m2: BDNF_S_LOG ~ Childhood_trauma + Timepoint + (1 | StudyID)
m3: BDNF_S_LOG ~ Childhood_trauma * Timepoint + (1 | StudyID)

   npar    AIC    BIC  logLik deviance  Chisq Df Pr(>Chisq)
m1    7 20.209 38.787 -3.1045   6.2091
m2    8 21.827 43.059 -2.9137   5.8274 0.3816  1     0.5367
m3   12 27.484 59.331 -1.7419   3.4837 2.3437  4     0.6728
```

The output shows that considering trauma type did not significantly improve the model or explain more of the data (values in last column are all greater than 0.05), suggesting that different trauma types do not explain the BDNF serum change.

VEGF-A serum by trauma type LMM output

Graph of the VEGF-A serum change by trauma type (childhood trauma vs other)

```
refitting model(s) with ML (instead of REML)
Data: df6
Models:
m1: VEGFA_S_LOG ~ Timepoint + (1 | StudyID)
m2: VEGFA_S_LOG ~ Childhood_trauma + Timepoint + (1 | StudyID)
m3: VEGFA_S_LOG ~ Childhood_trauma * Timepoint + (1 | StudyID)

   npar     AIC     BIC logLik deviance  Chisq Df Pr(>Chisq)
m1    7 -176.21 -157.64 95.107  -190.21
m2    8 -174.22 -152.99 95.111  -190.22 0.0089  1     0.9247
m3   12 -167.53 -135.69 95.767  -191.53 1.3112  4     0.8595
```

The output shows that considering trauma type did not significantly improve the model or explain more of the data (values in last column are all greater than 0.05), suggesting that different trauma types do not explain the VEGF-A serum change.

BDNF plasma by trauma type (no significant difference)

VEGF-A plasma by trauma type (no significant difference)

Serotonin by trauma type (no significant difference)

**Figure 2 – cytokine clusters in the trial by trauma type**

Participants with childhood trauma	Participants without childhood trauma

**Legend:** Clustering of cytokine levels by participant and study timepoint. P# indicate individual participants (rows). Values for each cytokine at each timepoint (columns) were normalised for comparison (subtract the group mean and divide by the group standard deviation – generates black for average values, green for values above the average and red for values below the average). White squares indicate missing samples. _Pre = pre-treatment sample, _Post = post-treatment sample, _F1W = follow-up 1 week sample, _F1M = follow-up 1 month sample.

Overall, there was no pattern in clustering by trauma type – both the “high” and “low” inflammatory groups contained childhood trauma participants.

Following up on the FKBP51 result from the main Figure 2 – When broken up by trauma type, there were not any statistically significant differences in FKBP51 between immune clusters within just the childhood trauma or other trauma groups (most likely because of small sample numbers).

**Figure 3 – Correlations between biomarkers and clinical scales by trauma type**

**Baseline correlations in participants with childhood trauma**

| row | column | cor | corrected p-values |
| --- | --- | --- | --- |
| IL1b_BA | IL2_BA | 0.944211 | 0.001796 |
| IL1b_BA | IL4_BA | 0.948333 | 0.001796 |
| IL12p70_BA | IL17A_BA | 0.953887 | 0.001796 |
| IL2_BA | IL4_BA | 0.903181 | 0.010654 |
| IL1b_BA | IL12p70_BA | 0.8997 | 0.010654 |
| IL2_BA | IL12p70_BA | 0.846732 | 0.038817 |
| IL1b_BA | TNFa_BA | 0.851068 | 0.038817 |
| Ser_BA | TNFa_BA | -0.82675 | 0.048049 |
| IL4_BA | TNFa_BA | 0.829268 | 0.048049 |
| PCL5_BA | DASS21_s_BA | 0.792201 | 0.049645 |

Significant correlations after multiple comparison correction.

**Baseline correlations in participants without childhood trauma**

| row | column | cor | corrected p-value |
| --- | --- | --- | --- |
| IL2_BA | IL17A_BA | 0.910396 | 0.001491 |
| IL12p70_BA | TNFa_BA | 0.904316 | 0.001491 |
| IL1b_BA | TNFa_BA | 0.887827 | 0.002091 |
| IL6_BA | TNFa_BA | 0.883721 | 0.002091 |
| IL1b_BA | IL6_BA | 0.859371 | 0.003239 |
| IL1b_BA | IL12p70_BA | 0.863091 | 0.003239 |
| IL6_BA | IL12p70_BA | 0.864752 | 0.003239 |
| IL4_BA | IL17A_BA | 0.817956 | 0.010775 |

Significant correlations after multiple comparison correction.

**Follow-up 1 correlations in participants with childhood trauma**

| row | column | cor | corrected p-value |
| --- | --- | --- | --- |
| IL1b_F1 | IL2_F1 | 0.963263 | 0.000305 |
| IL4_F1 | IL12p70_F1 | 0.963636 | 0.000305 |
| IL4_F1 | IL17A_F1 | 0.969223 | 0.000305 |
| PCL5_F1 | DASS21_d_F1 | 0.954133 | 0.00055 |
| IL12p70_F1 | IL17A_F1 | 0.944211 | 0.000951 |
| IL1b_F1 | IL17A_F1 | 0.919199 | 0.00338 |
| IL1b_F1 | IL4_F1 | 0.90303 | 0.00589 |
| IL1b_F1 | IL12p70_F1 | 0.890909 | 0.008132 |
| IL2_F1 | IL17A_F1 | 0.879905 | 0.010128 |
| DASS21_a_F1 | DASS21_s_F1 | 0.877636 | 0.010128 |
| IL2_F1 | IL4_F1 | 0.840554 | 0.024368 |
| IL2_F1 | TNFa_F1 | 0.838509 | 0.024368 |
| IL4_F1 | TNFa_F1 | 0.828283 | 0.028383 |
| IL17A_F1 | TNFa_F1 | 0.816603 | 0.031529 |
| DASS21_d_F1 | WHO5_F1 | -0.81709 | 0.031529 |
| BDNF_S_F1 | VEGFA_S_F1 | 0.806061 | 0.035884 |
| IL1b_F1 | TNFa_F1 | 0.803741 | 0.035884 |
| IL2_F1 | IL12p70_F1 | 0.791471 | 0.040276 |
| IL6_F1 | TNFa_F1 | 0.791471 | 0.040276 |
| PCL5_F1 | WHO5_F1 | -0.7842 | 0.043473 |
| PCL5_F1 | DASS21_s_F1 | 0.776762 | 0.046954 |

Significant correlations after multiple comparison correction.

**Follow-up 1 correlations in participants without childhood trauma**

| row | column | cor | corrected p-value |
| --- | --- | --- | --- |
| IL2_F1 | IL4_F1 | 0.980176 | 0.0000 |
| IL1b_F1 | IL2_F1 | 0.970298 | 0.0000 |
| IL1b_F1 | IL12p70_F1 | 0.964835 | 0.0000 |
| IL17A_F1 | PCL5_F1 | 0.969656 | 0.0000 |
| IL2_F1 | IL12p70_F1 | 0.943895 | 0.0000 |
| IL1b_F1 | IL4_F1 | 0.939495 | 0.0000 |
| IL12p70_F1 | TNFa_F1 | 0.934441 | 0.0000 |
| IL4_F1 | IL17A_F1 | 0.922575 | 0.0000 |
| IL4_F1 | IL12p70_F1 | 0.908691 | 0.0001 |
| IL2_F1 | IL17A_F1 | 0.907089 | 0.0001 |
| IL2_F1 | TNFa_F1 | 0.905401 | 0.0001 |
| IL4_F1 | TNFa_F1 | 0.903174 | 0.0001 |
| IL1b_F1 | TNFa_F1 | 0.898844 | 0.0001 |
| IL4_F1 | PCL5_F1 | 0.913343 | 0.0001 |
| PCL5_F1 | DASS21_d_F1 | 0.911143 | 0.0001 |
| IL17A_F1 | TNFa_F1 | 0.893756 | 0.0001 |
| IL12p70_F1 | IL17A_F1 | 0.883991 | 0.0002 |
| IL2_F1 | PCL5_F1 | 0.899449 | 0.0002 |
| IL1b_F1 | IL17A_F1 | 0.872942 | 0.0003 |
| IL6_F1 | IL12p70_F1 | 0.863143 | 0.0004 |
| TNFa_F1 | PCL5_F1 | 0.8809 | 0.0004 |
| IL1b_F1 | PCL5_F1 | 0.858323 | 0.0009 |
| IL1b_F1 | IL6_F1 | 0.827823 | 0.0013 |
| IL4_F1 | DASS21_a_F1 | 0.84584 | 0.0013 |
| IL4_F1 | WHO5_F1 | -0.8449 | 0.0013 |
| IL17A_F1 | DASS21_d_F1 | 0.838685 | 0.0016 |
| IL12p70_F1 | PCL5_F1 | 0.833563 | 0.0018 |
| IL2_F1 | WHO5_F1 | -0.82942 | 0.0019 |
| IL17A_F1 | WHO5_F1 | -0.8264 | 0.0021 |
| IL6_F1 | TNFa_F1 | 0.802259 | 0.0022 |
| IL17A_F1 | DASS21_a_F1 | 0.812596 | 0.0028 |
| IL2_F1 | DASS21_a_F1 | 0.807212 | 0.0032 |
| PCL5_F1 | WHO5_F1 | -0.80584 | 0.0032 |
| IL4_F1 | DASS21_d_F1 | 0.804476 | 0.0032 |
| TNFa_F1 | DASS21_d_F1 | 0.796092 | 0.0039 |
| PCL5_F1 | DASS21_a_F1 | 0.793001 | 0.0041 |
| IL2_F1 | DASS21_d_F1 | 0.780583 | 0.0053 |
| IL1b_F1 | WHO5_F1 | -0.76457 | 0.0074 |
| IL2_F1 | IL6_F1 | 0.735916 | 0.0083 |
| IL12p70_F1 | DASS21_a_F1 | 0.749335 | 0.0096 |
| IL4_F1 | IL6_F1 | 0.724866 | 0.0098 |
| IL1b_F1 | DASS21_a_F1 | 0.737981 | 0.0111 |
| DASS21_d_F1 | WHO5_F1 | -0.73846 | 0.0111 |
| IL1b_F1 | DASS21_d_F1 | 0.726803 | 0.0133 |
| IL6_F1 | IL17A_F1 | 0.694784 | 0.0155 |
| IL12p70_F1 | DASS21_d_F1 | 0.712932 | 0.0160 |
| TNFa_F1 | WHO5_F1 | -0.71269 | 0.0160 |
| DASS21_a_F1 | WHO5_F1 | -0.70693 | 0.0172 |
| IL6_F1 | DASS21_a_F1 | 0.702112 | 0.0183 |
| IL12p70_F1 | WHO5_F1 | -0.70086 | 0.0183 |
| TNFa_F1 | DASS21_a_F1 | 0.688799 | 0.0217 |
| DASS21_s_F1 | WHO5_F1 | -0.64615 | 0.0393 |
| IL17A_F1 | DASS21_s_F1 | 0.63423 | 0.0451 |
| PCL5_F1 | DASS21_s_F1 | 0.611132 | 0.0589 |
| IL6_F1 | PCL5_F1 | 0.603048 | 0.0635 |
| DASS21_a_F1 | DASS21_d_F1 | 0.600444 | 0.0643 |
| DASS21_d_F1 | DASS21_s_F1 | 0.581232 | 0.0784 |
| IL12p70_F1 | DASS21_s_F1 | 0.57423 | 0.0830 |
| IL4_F1 | DASS21_s_F1 | 0.565907 | 0.0891 |
| IL2_F1 | DASS21_s_F1 | 0.558353 | 0.0947 |
| IL6_F1 | WHO5_F1 | -0.55432 | 0.0970 |
| BDNF_S_F1 | IL1b_F1 | -0.51209 | 0.1184 |
| TNFa_F1 | DASS21_s_F1 | 0.525142 | 0.1245 |
| Ser_F1 | DASS21_s_F1 | -0.53809 | 0.1313 |
| IL1b_F1 | DASS21_s_F1 | 0.515974 | 0.1313 |
| FKBP51_F1 | DASS21_s_F1 | 0.526819 | 0.1744 |
| Ser_F1 | DASS21_a_F1 | -0.49745 | 0.1788 |
| DASS21_a_F1 | DASS21_s_F1 | 0.462872 | 0.1962 |
| VEGFA_S_F1 | IL6_F1 | 0.434883 | 0.2090 |
| BDNF_S_F1 | IL2_F1 | -0.40044 | 0.2673 |
| IL6_F1 | DASS21_d_F1 | 0.404467 | 0.2880 |
| Ser_F1 | FKBP51_F1 | -0.4022 | 0.3249 |
| BDNF_S_F1 | IL12p70_F1 | -0.35824 | 0.3427 |
| Ser_F1 | WHO5_F1 | 0.388714 | 0.3434 |
| VEGFA_S_F1 | DASS21_d_F1 | 0.355079 | 0.3741 |
| BDNF_S_F1 | IL6_F1 | -0.33334 | 0.3800 |
| VEGFA_S_F1 | IL12p70_F1 | 0.331868 | 0.3800 |
| IL6_F1 | DASS21_s_F1 | 0.345889 | 0.3800 |
| Ser_F1 | IL4_F1 | -0.33563 | 0.3983 |
| BDNF_S_F1 | WHO5_F1 | 0.332421 | 0.4007 |
| BDNF_S_F1 | VEGFA_S_F1 | 0.30989 | 0.4158 |
| BDNF_S_F1 | IL4_F1 | -0.30583 | 0.4158 |
| VEGFA_S_F1 | DASS21_a_F1 | 0.320738 | 0.4158 |
| Ser_F1 | IL1b_F1 | -0.3022 | 0.4509 |
| Ser_F1 | IL6_F1 | -0.29711 | 0.4515 |
| FKBP51_F1 | IL12p70_F1 | 0.309658 | 0.4515 |
| VEGFA_S_F1 | TNFa_F1 | 0.284782 | 0.4515 |
| VEGFA_S_F1 | PCL5_F1 | 0.283357 | 0.4748 |
| Ser_F1 | IL2_F1 | -0.28022 | 0.4770 |
| Ser_F1 | IL12p70_F1 | -0.26923 | 0.4771 |
| VEGFA_S_F1 | IL17A_F1 | 0.258567 | 0.4771 |
| BDNF_S_F1 | PCL5_F1 | -0.2696 | 0.4771 |
| Ser_F1 | PCL5_F1 | -0.28722 | 0.4771 |
| BDNF_S_F1 | DASS21_s_F1 | -0.27741 | 0.4771 |
| FKBP51_F1 | TNFa_F1 | 0.276492 | 0.4855 |
| VEGFA_S_F1 | IL1b_F1 | 0.248352 | 0.4882 |
| BDNF_S_F1 | TNFa_F1 | -0.24696 | 0.4882 |

Significant correlations after multiple comparison correction.

**Follow-up 2 correlations in participants with childhood trauma**

| row | column | cor | corrected p-value |
| --- | --- | --- | --- |
| IL12p70_F2 | IL17A_F2 | 0.979501 | 0.00002 |
| IL4_F2 | IL17A_F2 | 0.961278 | 0.00010 |
| IL1b_F2 | TNFa_F2 | 0.963343 | 0.00010 |
| IL4_F2 | IL12p70_F2 | 0.954545 | 0.00015 |
| IL1b_F2 | IL2_F2 | 0.936364 | 0.00053 |
| IL2_F2 | TNFa_F2 | 0.89912 | 0.00332 |
| IL12p70_F2 | TNFa_F2 | 0.862422 | 0.01089 |
| IL2_F2 | IL4_F2 | 0.836364 | 0.01231 |
| IL1b_F2 | IL12p70_F2 | 0.836364 | 0.01231 |
| IL4_F2 | TNFa_F2 | 0.844072 | 0.01231 |
| PCL5_F2 | DASS21_a_F2 | 0.846684 | 0.01231 |
| PCL5_F2 | DASS21_s_F2 | 0.853549 | 0.01231 |
| DASS21_a_F2 | DASS21_s_F2 | 0.841743 | 0.01231 |
| IL1b_F2 | IL4_F2 | 0.827273 | 0.01407 |
| PCL5_F2 | DASS21_d_F2 | 0.825307 | 0.01407 |
| IL17A_F2 | TNFa_F2 | 0.81841 | 0.01554 |
| IL1b_F2 | IL17A_F2 | 0.815492 | 0.01564 |
| IL2_F2 | IL17A_F2 | 0.80638 | 0.01810 |
| IL2_F2 | IL12p70_F2 | 0.790909 | 0.02366 |
| Ser_F2 | WHO5_F2 | 0.785396 | 0.02505 |
| DASS21_s_F2 | WHO5_F2 | -0.78211 | 0.02542 |
| DASS21_d_F2 | WHO5_F2 | -0.75807 | 0.03742 |
| Ser_F2 | TNFa_F2 | -0.75233 | 0.03943 |
| PCL5_F2 | WHO5_F2 | -0.73684 | 0.04844 |

Significant correlations after multiple comparison correction.

**Follow-up 2 correlations in participants without childhood trauma**

| row | column | cor | corrected p-value |
| --- | --- | --- | --- |
| IL1b_F2 | IL12p70_F2 | 0.944056 | 0.000118 |
| IL2_F2 | IL12p70_F2 | 0.949213 | 0.000118 |
| IL1b_F2 | IL17A_F2 | 0.952562 | 0.000118 |
| IL2_F2 | IL17A_F2 | 0.947189 | 0.000118 |
| IL1b_F2 | IL2_F2 | 0.9317 | 0.00025 |
| IL12p70_F2 | IL17A_F2 | 0.917412 | 0.000526 |
| IL2_F2 | IL4_F2 | 0.914187 | 0.000543 |
| IL4_F2 | IL17A_F2 | 0.903352 | 0.000751 |
| DASS21_a_F2 | DASS21_d_F2 | 0.903653 | 0.000751 |
| DASS21_s_F2 | WHO5_F2 | -0.86949 | 0.002863 |
| IL12p70_F2 | TNFa_F2 | 0.866219 | 0.002928 |
| IL4_F2 | IL12p70_F2 | 0.86014 | 0.003317 |
| IL1b_F2 | IL4_F2 | 0.853147 | 0.00386 |
| PCL5_F2 | DASS21_d_F2 | 0.844798 | 0.004656 |
| PCL5_F2 | DASS21_s_F2 | 0.83598 | 0.00564 |
| IL2_F2 | TNFa_F2 | 0.818354 | 0.008535 |
| FKBP51_F2 | IL2_F2 | 0.859856 | 0.009467 |
| IL4_F2 | TNFa_F2 | 0.809879 | 0.009467 |
| FKBP51_F2 | IL17A_F2 | 0.852106 | 0.009966 |
| DASS21_d_F2 | DASS21_s_F2 | 0.803887 | 0.009966 |
| PCL5_F2 | WHO5_F2 | -0.80282 | 0.009966 |
| DASS21_d_F2 | WHO5_F2 | -0.79542 | 0.010796 |
| FKBP51_F2 | IL12p70_F2 | 0.833996 | 0.014053 |
| DASS21_a_F2 | DASS21_s_F2 | 0.779264 | 0.014053 |
| IL1b_F2 | TNFa_F2 | 0.767625 | 0.017077 |
| DASS21_a_F2 | WHO5_F2 | -0.75233 | 0.021961 |
| FKBP51_F2 | IL1b_F2 | 0.782275 | 0.033282 |
| IL17A_F2 | TNFa_F2 | 0.704426 | 0.043606 |
| PCL5_F2 | DASS21_a_F2 | 0.706675 | 0.043606 |
| IL6_F2 | WHO5_F2 | 0.721469 | 0.048821 |

Significant correlations after multiple comparison correction.
